# Sex differences in obesity related cancer incidence in relation to type 2 diabetes diagnosis (ZODIAC-49)

**DOI:** 10.1371/journal.pone.0190870

**Published:** 2018-01-25

**Authors:** Dennis Schrijnders, Steven H. Hendriks, Nanne Kleefstra, Pauline A. J. Vissers, Jeffrey A. Johnson, Geertruida H. de Bock, Henk J. G. Bilo, Gijs W. D. Landman

**Affiliations:** 1 Langerhans Medical Research Group, Zwolle, the Netherlands; 2 Diabetes Centre, Zwolle, the Netherlands; 3 University of Groningen, University Medical Center Groningen, Department of Internal Medicine, Groningen, the Netherlands; 4 Netherlands Comprehensive Cancer Organization, Utrecht, the Netherlands; 5 School of Public Health, University of Alberta, Edmonton, Canada; 6 University of Groningen, University Medical Center Groningen, Department of Epidemiology, Groningen, the Netherlands; 7 Department of Internal Medicine, Zwolle, the Netherlands; 8 Department of Internal Medicine, Gelre Hospital, Apeldoorn, the Netherlands; University of Glasgow, UNITED KINGDOM

## Abstract

**Background:**

Diabetes and obesity seem to be partly overlapping risk factors for the development of obesity-related cancer (mainly breast, prostate and colorectal cancer) in patients with type 2 diabetes (T2DM). In the general population, women have a lower risk for obesity-related cancer compared to men. Previous studies involving cardiovascular disease have shown that T2DM eliminates a female advantage of lower CVD risk in the general population compared to men. It is unclear whether the same could be true for obesity-related cancer. This study aimed to this investigate obesity-related cancer incidence in women and men known with T2DM as compared to the Dutch general population.

**Methods:**

This study included 69,583 patients with T2DM selected from a prospective primary care cohort, which was linked to the Dutch National Cancer Registry to obtain cancer specific data. Obesity-related cancers included liver, kidney, colorectal, gallbladder, pancreas, ovarian, endometrial, advanced prostate cancer, post-menopausal breast cancer and oesophageal adenocarcinoma. Primary outcome was sex-stratified, age and year of cancer diagnosis adjusted standardized incidence ratios (SIRs) for three time periods: 5 years before, the year after diagnosis and the next 4 years after T2DM diagnosis. The Dutch general population was used as reference group.

**Results:**

Women with T2DM were at an increased risk for obesity-related cancer compared to women in the general population already 5 years before diabetes diagnosis (SIR 1.77; 95%CI: 1.63–1.91). In both men and women, there was a peak in obesity-related cancer incidence following diabetes diagnosis (SIR: 1.38; 95%CI 1.11–1.64 and SIR: 2.21; 95%CI 1.94–2.30, respectively). From the second to the fifth year after diabetes diagnosis the obesity-related cancer incidence was higher in women compared to women in the general population (SIR: 2.12; 95%CI 1.94–2.30).

**Conclusions:**

Women with T2DM seem to have a substantially higher obesity-related cancer risk. As opposed to men, in women this risk was already increased years before diabetes diagnosis. These results could imply that a relative advantage of women in the general population with regard to cancer risk is lost in women with T2DM.

## Introduction

Patients with type 2 diabetes (T2DM) have an increased risk of developing specific types of cancer compared to the general population[1]. An increased risk has been reported for the development of cancers of the liver, pancreas, endometrium, colon, rectum, breast and bladder[1–4]. The increased risk of these specific types of cancer in patients with T2DM largely overlap with the so called ‘obesity-related cancers’. The World Cancer Research Fund acknowledges liver, kidney, colorectal, gallbladder, pancreas, ovarian, endometrial, advanced prostate cancer, post-menopausal breast cancer and oesophageal adenocarcinoma as obesity-related cancer[4, 5]. A large portion of patients with T2DM are overweight or obese[6, 7]. So, diabetes and obesity appear to be partly overlapping risk factors for the development of cancer in patients with T2DM[8, 9]. The development of diabetes is mostly a progressive process[10]. It is well-known that being overweight is a very important factor in type 2 diabetes[11]. Patients who are diagnosed with T2DM are already different with respect to i.e. body mass index, compared to persons in the general population also in the years prior to diabetes diagnosis[12, 13]. Furthermore, these patients will for example have a more frequent impaired glucose tolerance and/or impaired fasting glucose, accompanied by higher glucose and insulin levels[14, 15]. Both are growth factors important in tumour development[16, 17]. In addition, it has been suggested that sex hormones increases cancer risk[18].

In the general population, risk for obesity-related cancer, like the risk for cardiovascular disease (CVD), is lower in women than in men[19]. However, previous studies investigating risk of CVD have shown that T2DM eliminates this female advantage[20, 21]. It is unclear if the same could be true for (obesity-related) cancer. Indeed, several small studies reported sex differences in cancer risk in patients with pre-diabetes or T2DM. Reports show that in the pre-diabetes phase, the risk to develop breast cancer is increased compared to women without pre-diabetes [22, 23]. In addition, a significant increase in colon cancer incidence before T2DM diagnosis has been reported in men, where this was not observed in women[24, 25]. The increased incidence of cancer among T2DM patients has only partly been observed in larger studies that investigated the distribution of overall incidence of cancer over the years before and after the diagnosis of diabetes[2, 26]. However, those studies did not stratify the risk to develop cancer for males and females[2, 26]. T2DM and adiposity have been associated with a significant additive effect of obesity-related cancer[27]. This increased cancer incidence seems to be different in the periods before the diagnosis of diabetes, shortly after (1 year) and the period after 1 year after the diagnosis of diabetes[28]. Thus far, only few studies investigated the overall and obesity-related cancer risk in T2DM stratified for males and females to answer the question whether there are sex differences in cancer risk.

The primary aim of this study was to investigate the incidence of obesity-related cancers in women and men in relationship to the time of diabetes diagnosis. The obesity-related cancer incidence was analysed in the periods 5 years before, the year after and 2 to 5 years after the diagnosis of diabetes. A secondary aim was to investigate the incidence of all-cancer incidence in patients with a diagnosis of diabetes.

## Methods

This study is reported according to the STROBE (Strengthening the reporting of observational studies in epidemiology) recommendations[29].

### Study design and data collection

For this study, two prospective cohorts were combined. A T2DM specific cohort, the observational ZODIAC (Zwolle Outpatient Diabetes project Integrating Available Care) cohort study, was merged with the Netherlands Cancer Registry (NCR). To obtain cancer specific data for patients included in the ZODIAC cohort, all cancer events that occurred between 1989 and 2012 were linked to the data of the ZODIAC cohort via a trusted third party using postal code, full name, date of birth and sex. The NCR expects that the number of false-positive and the number false-negative for the ZODIAC-NCR linkage is both under 1%.

ZODIAC is a prospective primary care cohort study, which was initiated in 1998 in the Zwolle region. The cohort has been expanded to different regions several times and now includes almost all patients in the north-eastern part of the Netherlands, part of Flevoland province and part of the province of North Holland. As such, the cohort represents the majority of primary care treated patients in the Netherlands and has a high degree of generalizability. In the ZODIAC study, clinical data were sent annually to the Diabetes Centre by general practitioners (GPs) for benchmarking and research purposes. Patients included in the ZODIAC study were diagnosed with T2DM and were at inclusion treated exclusively in primary care. There was no age restriction for participation. Patients were not eligible for participation if they had a short life expectancy, a cognitive impairment or were treated in secondary care. From all patients included in ZODIAC, diabetes specific data was electronically collected annually. Data includes date of birth, sex, date of diabetes diagnosis, HbA1c, height, weight, serum creatinine, albuminuria, cholesterol/HDL ratio, blood pressure, micro- and macrovascular complications, medication use (both diabetes specific and other medication), smoking status and alcohol use. Entire primary care practises participated in ZODIAC and the project has been expanded to new regions several times and therefore included newly diagnosed and previously diagnosed T2DM patients. All patients were followed prospectively. All T2DM patients within participating primary care practises who were treated in primary care for their diabetes could participate.

The primary care provider in the Netherlands has a very important role in the health care system, for example as a mandatory gatekeeper to secondary care. All inhabitants of the Netherlands have a GP and health insurance. Since the late nineties the majority of patients (around 88%) with T2DM are treated exclusively in primary care[30] and benchmarking has become a mandatory part of receiving health care reimbursements. The quality of the Dutch diabetes primary care is regarded to be high and for example the average HbA1c is 6.7%[6].

The NCR is a population based mandatory national registry, founded in 1989, and records all malignancies based on notification by the National Pathology Archive (PALGA) and hospital discharge registries. Data is gathered by specially trained data-managers directly from the patients’ files in hospitals in the Netherlands. Data recorded includes incidence date, TNM stage, morphology, location and primary cancer treatment. Basal cell carcinoma of the skin, carcinoma in situ of the cervix, myelodysplastic syndrome, myeloproliferative disorders were not registered in the NCR database. Benign and borderline tumours were excluded, with the following exceptions; benign brain tumours (included from 1999), carcinoids of the appendix (included from 2001), borderline tumours of the ovaries (included from 2001), thymoma (included from 2001), phyloides tumours (included from 2001) and T-cell leukaemia (included from 2004). Comorbidities, including diabetes, were also not registered in this database.

### Patient selection

The combined ZODIAC-NCR database contained 71,648 patients of which 10,717 were diagnosed with 12,617 cancer events between 1 January 1998 and 31 December 2012. Only the first obesity-related cancer event (for the obesity-related cancer analyses) or first cancer event (in the all-cancers combined analyses) were included in the analysis. From the 71,648, a total of 2,065 (2.9%) patients were excluded because a cancer event occurred before the study period of interest, i.e. more than 5 years before diabetes diagnosis.

Baseline (time zero) was set at the date of diabetes diagnosis. The date of diabetes diagnosis was based on the electronically documented date of diabetes diagnosis derived from the medical history for patients with newly diagnosed diabetes. As previously mentioned, the date of diabetes diagnosis could very well be different to the time of entry into the ZODIAC study.

### Outcome measures

The primary outcome was the sex-stratified, age and year of cancer diagnosis adjusted standardized incidence ratios (SIR) of obesity-related cancers in three time frames; the 5 years before diabetes diagnosis, the first year following diabetes diagnosis and year 2 to 5 after diabetes diagnosis. The comparator group was the Dutch general population (including patients with diabetes). The following cancers (ICD-10 codes) were defined as obesity-related cancer: liver (C22), kidney (C64), colorectal (C18, C19, C20), gallbladder (C23), pancreas (C25), ovarian (C56), endometrial (54.1) and advanced prostate cancer (C61 with TNM 3 or 4 or Gleason > 7), post-menopausal breast cancer (C50 and age > 54) and oesophageal adenocarcinoma (C15.5)[5].

The secondary outcome was the same as the primary outcome but, instead of obesity-related cancer, combined all-cancers (non-melanoma skin cancer were excluded).

### Statistical analysis

Using the date of diabetes diagnosis and the date of the first cancer event, age and year of cancer diagnosis standardized obesity-related and overall cancer incidence were calculated for one-year intervals from 5 years prior to 5 years after the diagnosis of T2DM.

Patients from whom only the year (and not the month) of diabetes diagnosis was available, the first of July was chosen as date of diabetes diagnosis.

The cancer incidence rate in the ZODIAC cohort was calculated first. The NCR provided incidence rates of the general population from the same time points that were included in the ZODIAC cohort. The NCR is unable to identify diabetes status, and thus the comparator group also includes patients with diabetes. Standardized incidence ratios with 95% confidence intervals stratified for sex and adjusted for age and year of cancer (in 5-year intervals) were calculated to compare the cancer incidence in the ZODIAC cohort to the general population[31]. The SIR is analogous to the commonly used standardized mortality ratios[32]. Incidence ratios were calculated by dividing the total number of cancer events in one-year time periods by the amount of contributed time in this one-year time period. Reference data for all-cancer and for obesity-related cancer incidence of the whole Dutch population were provided by the NCR.

Both all-cancer and obesity-related SIR were stratified for sex. A separate analysis stratifying by BMI was performed. This analysis only includes patients who have a BMI recorded at diabetes diagnosis. Advanced prostate cancer and post-menopausal breast cancer were also examined separately. Furthermore, to make obesity-related cancers somewhat comparable in men and women T2DM an analysis excluding sex-specific cancers was performed. Sex-specific obesity-related cancers were ovarian, endometrial and post-menopausal breast cancer in women and advanced prostate cancer in men.

A sensitivity analysis excluding pancreas cancer was performed to investigate the effect of pancreatic cancer on T2DM diagnosis. Analyses were performed in STATA v14.

### Ethics statement

The medical ethics committee of Isala, Zwolle, the Netherlands approved the procedure of this study including the anonymous linking of the ZODIAC with the NCR (METC reference number 13.0765). No written informed consent was required. Patients consented with the anonymous use of their data for study purposes in the ZODIAC study.

## Results

Baseline characteristics are shown in Table 1. Among the 69,583 patients included, 49% were female and 40,417 patients had a follow-up of at least 5 years. Women were older and had a higher BMI compared to men.

**Table 1:** Patient characteristics.

	Women	Men	P-value *
	(n = 34,312, 49.5%)	(n = 35,271, 50.5%)	
Age at diabetes diagnosis (SD), years	63.0 (12)	605 (11)	< 0.001
Diabetes duration (IQR), years	2.5 (0.6–6.3)	2.1 (0.5–5.7)	
HbA1c at diabetes diagnosis (IQR), mmol/mol	49 (43–55)	49 (43–55)	0.32
HbA1c at diabetes diagnosis (IQR), %	6.6 (6.1–7.2)	6.6 (6.1–7.2)	
BMI at diabetes diagnosis§ (IQR)	29.5 (26.3–33.7)	28.4 (26.0–31.5)	< 0.001
Creatinine at diabetes diagnosis (IQR), μmol/L	68 (59–80)	87 (74–95)	< 0.001
Cancer events			
All-cancer (n, %)	2130 (6.2)	2139 (6.1)	
Obesity-related cancer (n, %)	1344 (3.9)	709 (2.0)	
Non sex-specific obesity-related cancer (n, %)	374 (1.1)	479 (1.4)	
BMI categories§			< 0.001
18 to 25 (%)	16.5	16.8	
25 to 30 (%)	36.6	47.1	
>30 (%)	46.9	36.1	

Continuous data were analysed using independent t-tests or the Mann-Whitney U test. Categorical variables were analysed using chi square tests.

SD: Standard deviation, IQR: Inter-quartile range, BMI: Body mass index.

^§^ Includes only patients who had a BMI recorded at diabetes diagnosis.

*P-value for the difference between men and women at baseline.

### Obesity-related cancers

**Fig 1:**
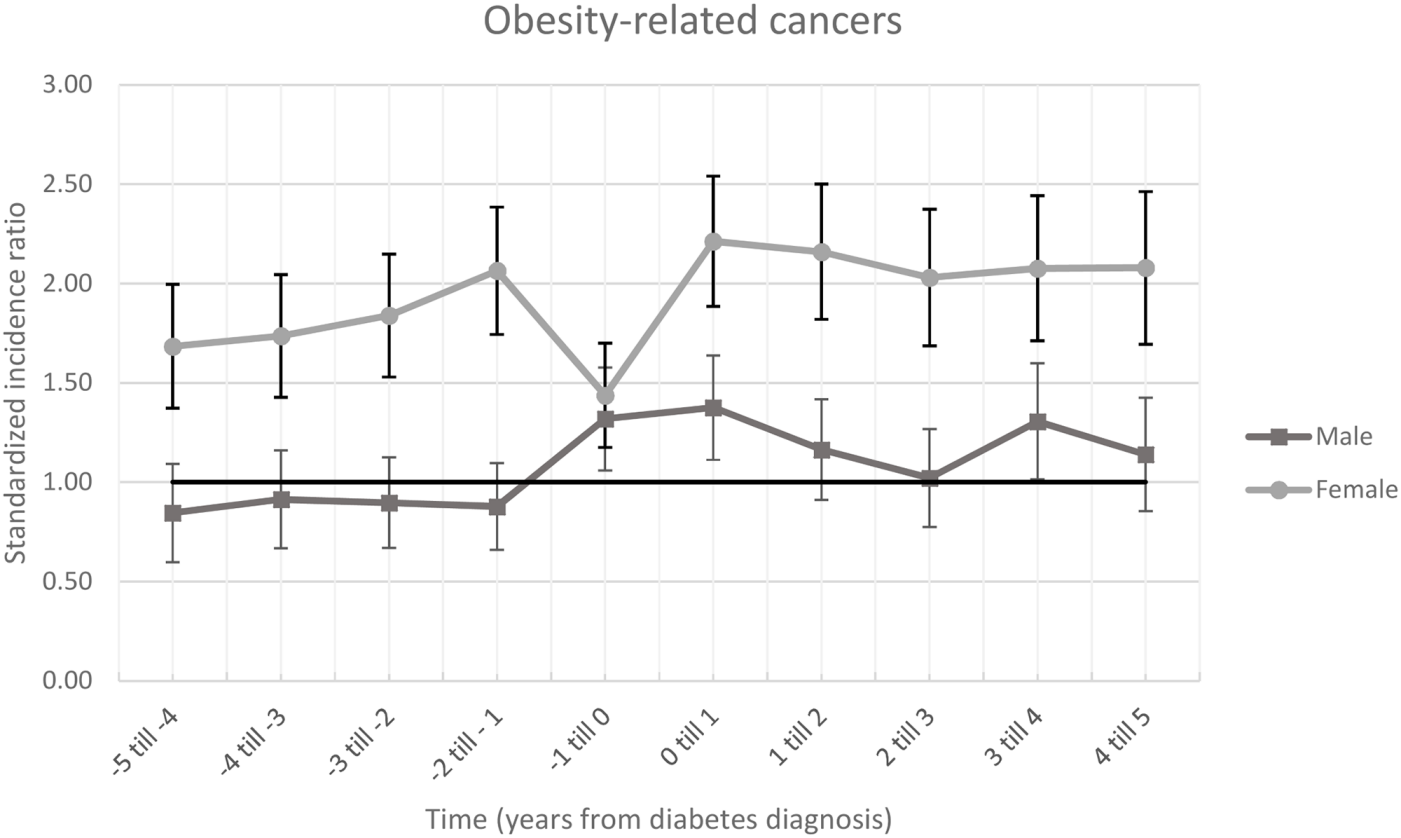
Standardized incidence ratio with 95%CI bars of obesity-related cancer. The horizontal line is 1.00 and means that there was no difference between the ZODIAC population and the general population.

Incidence ratios for obesity-related cancers are shown in Fig 1, S1 and S2 Tables. Compared to the general population, in men and women combined the incidence of obesity-related cancer were elevated in the 5 years prior to, the first year after and year 2 to 5 after diabetes diagnosis, with the highest cancer incidence in the first year after diabetes diagnosis (SIR 1.80, 95%CI 1.59–2.01).

In women, the 5 years prior to diabetes diagnosis, there was a significantly increased obesity-related cancer incidence in all years compared to women in the general population. The SIR gradually increased from 1.86 (95%CI 1.37–2.00) at 5 years prior to diabetes diagnosis to 2.06 (95%CI 1.74–2.39) at 2 to 1 year prior to diabetes diagnosis but decreases in the year before diagnosis of diabetes (SIR 1.44, 95%CI 1.17–1.70). The first year after diabetes diagnosis the SIR was the highest (SIR 2.21, 95%CI 1.88–2.54) and remained increased in the 5 years after diabetes diagnosis.

In men, no significantly increased cancer risk was observed in 5 to 1 year prior to diagnosis of diabetes. In contrast, in the year prior and the year after diabetes diagnosis the SIR was significantly increased cancer risk compared to men in the general population, 1.32 (95%CI 1.06–1.58) and 1.38 (95%CI 1.11–1.64), respectively.

An analysis where patient were stratified by BMI below or above 30 at diabetes diagnosis were in line with the main analyses (S7 Table and S3 and S4 Figs). The SIR for obesity-related cancers was higher in women compared to men for all years, with non-overlapping confidence intervals, except the one year before diagnosis of diabetes.

### Sex-specific influences on obesity-related cancer incidence

**Fig 2:**
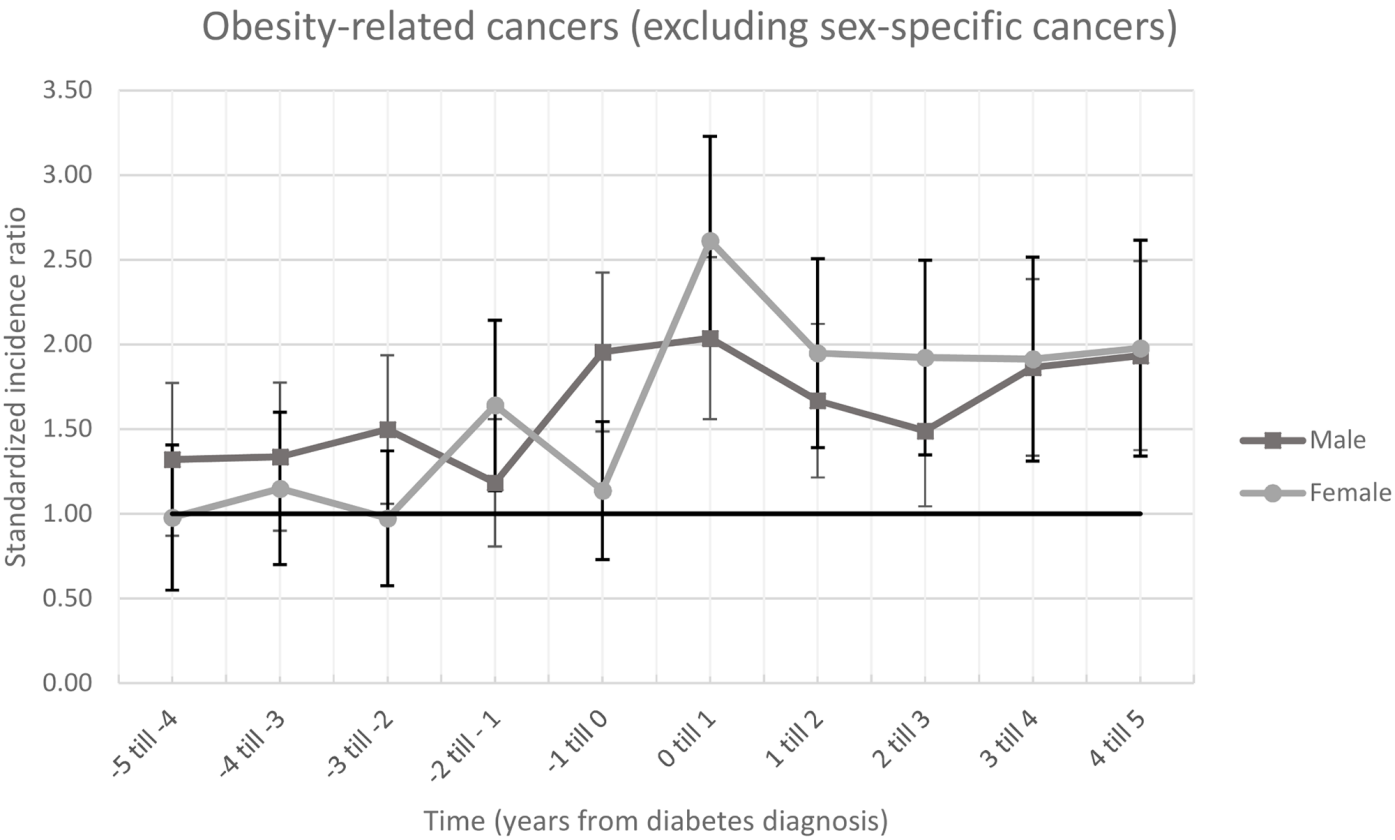
Standardized incidence ratio with 95%CI bars of obesity-related cancer excluding sex-specific cancers. The horizontal line is 1.00 and means that the was no differences between the ZODIAC population and the general population.

Fig 2, S1 and S3 Tables show the SIR for obesity-related cancer when the sex-specific obesity-related cancer types (breast, ovarian and endometrial cancer in women and advanced prostate cancer in men) were excluded. In women a significantly increased SIR for obesity-related non-sex specific cancer before diabetes diagnosis was only observed for year minus 2 to minus 1 year before diabetes diagnosis (SIR 1.64, 95%CI 1.14–2.14). In the year following diabetes diagnosis a significantly increased SIR was observed (SIR 2.61, 95%CI 2.00–3.23) for all years after diabetes diagnosis. A separate analysis which included only breast cancer (S6 Table and S1 Fig) showed that the SIR was increased for all time points, and also showed an apparent decrease the year before diabetes diagnosis.

In men, there was a significant difference in cancer incidence at minus 3 years (SIR 1.50, 95%CI 1.06–1.94) and minus 1 year (SIR 1.96, 95%CI 1.49–2.42) prior to diabetes diagnosis compared to the general population. Cancer incidence was highest in the first year after diabetes diagnosis (SIR 2.04, 95%CI 1.56–2.51). A separate analysis investigating specifically advanced prostate cancer showed a decreased incidence in advance prostate cancer the 5 year prior to diabetes diagnosis and no significant difference compared to the general population in the years after diabetes diagnosis.

The SIRs for non-sex-specific obesity-related cancers were, based on overlapping confidence intervals, not significantly different between sexes.

### Standardized incidence ratios—All-cancers combined

**Fig 3:**
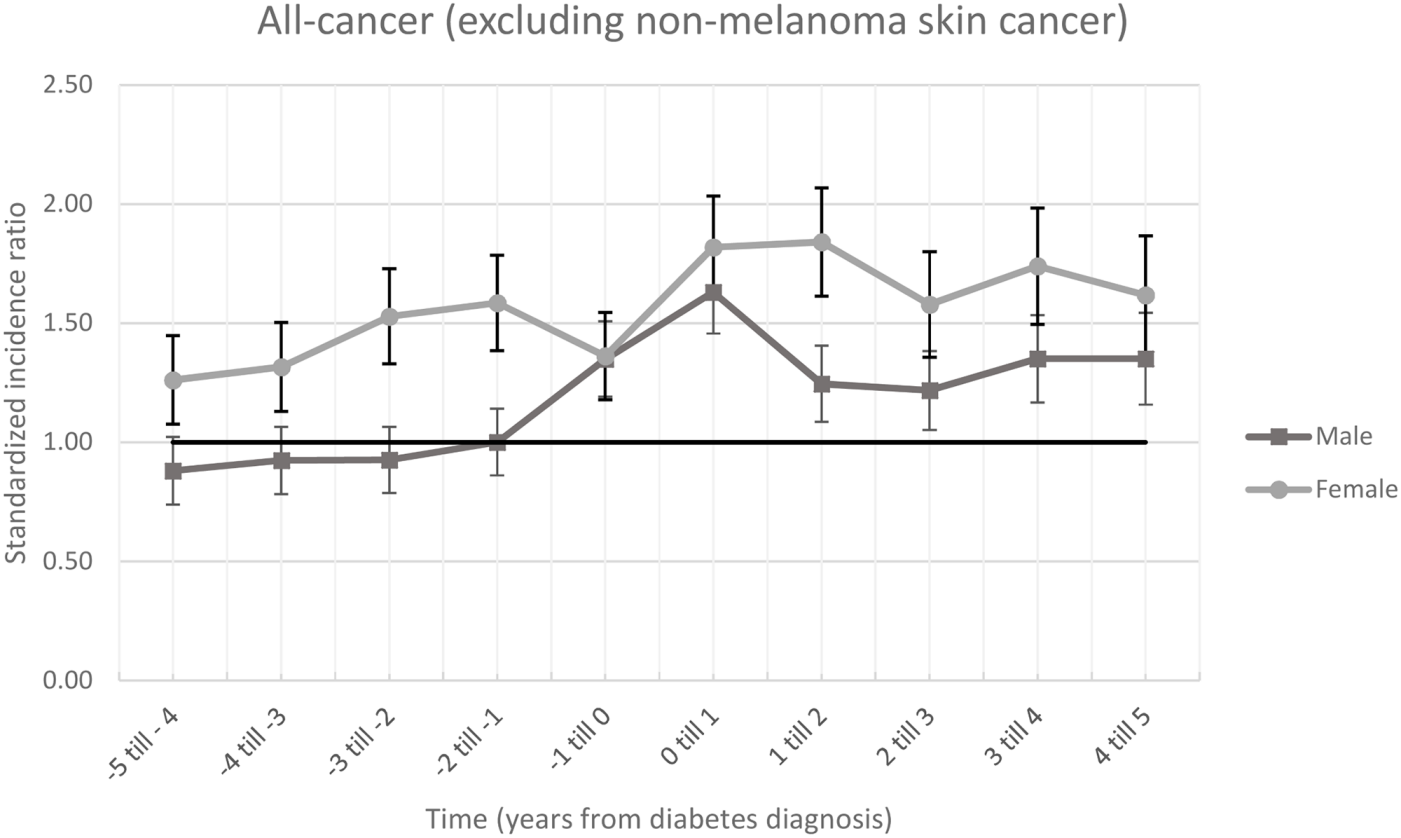
Standardized incidence ratio with 95%CI bars of all-cancer combined (excluding non-melanoma skin cancer). The horizontal line is 1.00 and means that the was no differences between the ZODIAC population and the general population.

The SIR for all-cancers combined for men and for women are shown in Fig 3, and S1 and S4 Tables. Over the years, there was a gradual increase in SIR in men and women combined, becoming significantly from 3 to 2 years prior to diagnosis of diabetes (see S4 Table). A rise in cancer incidence was present for men and women combined the year prior to and around diabetes diagnosis, see S4 Table. There were increased cancer incidences in year 2 to 5 after diabetes diagnosis.

In women, there was an increased cancer incidence that started to increase from 5 years prior to diabetes diagnosis, see Fig 3 and S4 Table. In men, only the year prior to diabetes diagnosis and 5 years thereafter were increased, see Fig 3 and S4 Table. A sensitivity analysis excluding pancreas cancer did not change results significantly (S5 Table). An analysis where patient were stratified by a BMI below or above 30 at diabetes diagnosis were in line with in line with the main analyses (S8 Table and S3 and S4 Figs).

## Discussion

This study is the first to investigate standardized obesity-related cancer incidence ratios in three time periods (5 years before, the first year after and 2 to 5 years after diabetes diagnosis) and accounting for sex-differences.

In women there was an increase in obesity-related cancer incidence already 5 years before T2DM diagnosis and this was also increased after diabetes diagnosis compared to women in the general population. This was not observed in men. Also from year 2 to 5 after diabetes diagnosis, women had a higher obesity-related cancer incidence compared to women in the general population.

The observed increase in obesity-related cancer incidence in women with T2DM compared to women in general population was explained to a large part by the contribution of sex-specific cancers, for example breast cancer. The exclusion of sex-specific cancers in women mitigated the increased obesity-related cancer incidence before diabetes diagnosis, highlighting the importance of sex-specific cancers in women. Furthermore, the differential effects do not seem to be confounded by obesity. It seems to be that after diabetes diagnosis, irrespective of the presence of obesity or not, there was an increased cancer incidence compared to the general population.

The analysis that included only breast cancer was in line with the obesity-related and all-cancer analyses and showed an increased breast cancer incidence in women with diabetes compared to the general population. This is in accordance with previous studies that investigated breast cancer incidence also showed that breast cancer risk was already elevated in the pre-diabetes phase[23, 33]. It could be that women put on relative more weight in the pre-diabetes phase. From all obesity-related cancers, breast cancer appears to show the strongest correlation with weight increases [10, 34]. It has been suggested that the increased incidence could be due to increased aromatization of androgens to oestrogens locally in fat tissue[35]. Eventually, this could lead to increased ERα signalling in breast cancer and possibly a greater risk of oestrogen-dependent breast cancer[35]. Indeed, especially oestrogen receptor positive breast cancer risk has been reported to be increased in women with T2DM[36].

There was a decrease in obesity-related SIR in women 1 year before diabetes diagnosis for which we have no obvious explanation. There is some evidence that especially breast cancer in strongly associated with obesity and weight gain as such[37, 38]. There are reports suggesting that obese patients could also be less likely to respond to screening programs[38, 39]. During the study period, there were no national screening programs involving men. Another possible explanation could be related to the care provided. It could be that there is some degree of clinical distraction or pre-occupation with metabolic parameters that results in fewer diagnosis of cancer[40]. An incidental cancer diagnosis is perhaps delayed until the diagnosis of diabetes is made. It could also be that during the year prior to diagnosis of diabetes cancer screening tests are performed but the definitive cancer diagnostic test take longer to complete, thus delaying the cancer diagnosis until after diabetes is diagnosed. However, most cancer diagnostic tests don’t take long to complete. A fourth possible explanation is that there is an issue with the registration of cancer events. However, this seemed unlikely since all cancer diagnoses in the Netherlands are automatically registered with the Cancer Registry. None of these factors seemed plausible. A final explanation could be that this finding is a coincidence for which we have no causal explanation. Cancer screening programmes in place in the Netherlands at the time of the study period are for breast cancer and cervical cancer. Nationwide, there was an attendance rate of 79% and 65%, respectively[41, 42]. Previous studies have shown that women with comorbidity, for example obesity[39], and lower social economic status are less likely to attend screening[43–45]. The incidence of diabetes is increased in people with lower socio-economic status[46–48]. It would thus be more likely that there would be a decreased screening attendance instead of an increased screening attendance in the prediabetes phase. In addition, women with diabetes are also less likely to undergo a mammogram than women without diabetes[43]. Some studies have shown more favourable survival rate in screen detected breast cancers and emphasized the importance of cancer screening in patients with diabetes[49]. In the analyses excluding sex-specific cancers this decline disappeared, which could point to breast cancer as a likely causative cancer. The decline was not present in men, which likely excludes a methodological error as an explanation. Overall, we have no causal explanation for this finding.

There was a pronounced increase in cancer incidence around the time of diabetes diagnosis. This increased detection has been reported in previous studies [26, 50]. However, it seems questionable that detection bias is an important plausible explanation before diabetes diagnosis and in the years after the first year following diabetes diagnosis[2, 26, 28, 49]. Another possible explanation for the differential effects observed is diagnostic bias. Patients diagnosed with diabetes are more likely to have additional diagnostic tests[50–52]. The first year after diabetes diagnosis, obesity-related cancer incidence increased 121% for women and 38% for men compared to the general population. No previous studies combining obesity-related cancer incidence were found. However, an Austrian cohort study (n = 5709, T2DM patients) also reported elevated SIRs for cancers of the pancreas (1.78, 95%CI 1.02–2.89) and corpus uteri (1.79, 95%CI 1.15–2.66) in women and for cancers of the liver (2.71, 95%CI 1.65–4.18) and pancreas (1.87, 95%CI 1.11–2.96) in men in the year after diagnosis of diabetes[53].

The peak in cancer incidence around diabetes diagnosis could partly be directly explained by diabetes developing as a result of for example pancreas cancer[49, 51]. A higher cancer mortality has been reported in female pancreatic cancer patients compared to men[54]. However, exclusion of pancreas cancer in a sensitivity analyses did not relevantly change results (S5 Table). Increased detection of diabetes in a cancer work-up or cancer in a diabetes work-up could also play a role in the rise in cancer incidence immediately after diagnosis of diabetes in both men and women[50–52]. There is evidence from previous studies that detection bias is partly responsible for the increase in cancer incidence patients with T2DM, at least for the excessive peak that has been observed [2, 26].

In the period from 2 to 5 years after diabetes diagnosis obesity-related and all-cancer incidence was substantially and significantly higher in women and remained increased throughout year 2 to 5 after diabetes diagnosis. In men, there was a less obvious increase in obesity-related and all-cancer incidence.

In men, as opposed to women, exclusion of sex-specific obesity-related cancer (i.e. advanced prostate cancer) substantially increased obesity-related cancer incidence the years after diabetes diagnosis. This could point to a protective effect of T2DM to prostate cancer. Previous meta-analyses already showed a decreased risk of low stage prostate cancer in T2DM patients[49, 55, 56]. The analysis which included only advanced prostate cancer showed a decreased cancer incidence in the 5 years prior to diabetes diagnosis, and no difference in cancer risk after diabetes diagnosis. Our results could suggest that advanced prostate cancer might thus not be positively related to obesity. These result are in contrast to the definition used by the WCRF[5]. A recent viewpoint of the International Agency for the Research of Cancer (IARC) also stated that there is limited evidence for fatal prostate cancer being related to obesity[57].

### Strengths and limitations

This study sample represents the majority of primary care treated T2DM patients in the Netherlands and has a high degree of generalizability. Another strength of this study was the size of the ZODIAC cohort. Thirdly, the long follow-up periods made it possible to estimate incidence ratios as early as five years before diagnosis of diabetes and study cancer incidence before diabetes onset.

There were also limitations. Some patients were selected with a date of diabetes diagnosis occurring before 1994. In theory, taking into account the 5 year window before diabetes diagnosis used in this study, patients could already have had a cancer event while diagnosed with diabetes before the NCR commenced in 1989. This cancer event would not have been registered in the NCR. However we expect this effect to be fairly negligible, since only 4.4% of patients in our data were diagnosed with diabetes before 1994. A second limitation was that cancer incidence before diabetes diagnosis could be influenced by survival bias. Since the ZODIAC cohort only consists of patients diagnosed with T2DM, patients who died from, amongst others, cancer, could never have been included in this cohort since they did not have time to develop diabetes. Thirdly, the general population also included people diagnosed with diabetes; around 10% of the general population in this age category in the Netherlands are diagnosed with diabetes[58, 59]. It has been shown that with increasing age the proportion of people diagnosed with diabetes increases[59]. Furthermore, as opposed to our cohort, the general population also included people with a history of cancer. If these two factors would be accounted for they both would likely lead to an underestimation of the effect seen. The reported higher cancer incidence rates in patients with diabetes would in reality be more pronounced[58]. In addition, we were unable to adjust for confounders other than age and sex. For example, BMI data in the general population were not available. Fourthly, the ZODIAC cohort expanded to different regions in the Netherlands at several time points and both patients with known diabetes and newly diagnosed diabetes were included. The date of diabetes diagnosis was based on an electronic health record and verified by a practice nurse. This could have been subject to recall bias. For example some patients could not remember the month of diabetes diagnosis. In this case the first of July was used as month of diabetes diagnosis in an attempt to minimize this bias. Unfortunately, we could not exclude a relevant effect of this recall bias, however, we have no reason to assume that this would be different in men or women.

It is plausible that detection bias is also present in the current study, explaining the peak in cancer incidence at diabetes diagnosis. This could in turn also explain the slight decrease in cancer risk seen in the 2 to 3 years after diabetes diagnosis in the present study which would be almost absent if there would not have been a peak. Increased detection does not necessarily lead to bias nor is it always a negative effect, but it is a phenomenon that partly results from increased contact with health care professionals[26]. Although no specific indications exist which suggests that people in the ZODIAC regions have a different cancer risk compared to the general population in the Netherlands, we do not know that for sure. It would have been better if we could have used a comparator group from the ZODIAC regions, but unfortunately such a group was not available. Lastly, hormone receptor status of breast cancer was not available in the current study, making it impossible to investigate the influence of oestrogen-dependent breast cancer. This will be subject for further studies.

In summary, in men and women with T2DM, cancer incidence peaked the year after diabetes diagnosis. A clear difference in cancer risk was observed between men and women. Women, not men, with T2DM had an increased obesity-related standardised cancer incidence compared to women in the general population, as early as 5 years before diabetes diagnosis. After diabetes diagnosis, both men and women had an increased SIR but the effect was more pronounced in women.

Exclusion of sex-specific obesity-related cancers resulted in a lower incidence in women and a higher incidence in men compared to the SIR in all obesity-related cancers. The latter might suggest that, in addition to low risk prostate cancer, advanced prostate cancer is not associated with obesity in patients diagnosed with T2DM.

Next to relative cancer risk, absolute obesity-related cancer risk in the general population show that women have a lower risk than men[19]. It appears to be that the relative advantage in women disappears in the presence of diabetes and exemplify that the relative protective effect in women is lost when diabetes develops, which is comparable to CVD. The difference in women with T2DM compared to women in the general population and the difference of women compared to men with T2DM could mean that in the general population women have an advantage concerning cancer risk but when diabetes develops this advantage disappears. The impact of this should be subject of further investigations.

## Supporting information

S1 Table Pooled standardized incidence ratio. (DOCX)

S2 Table Standardized incidence ratio of obesity-related cancers. Cancers included: liver, kidney, colorectal, gallbladder, pancreas, ovarian, endometrial and advanced prostate cancer, post-menopausal breast cancer and esophageal adenocarcinoma.
(DOCX)

S3 Table Standardized incidence ratio of obesity-related cancer excluding sex-specific cancers. Cancers included: liver, kidney, colorectal, gallbladder, pancreas and esophageal adenocarcinoma.
(DOCX)

S4 Table Standardized incidence ratio all cancers combined. (DOCX)

S5 Table Standardized incidence ratio all cancers combined (excluding pancreas). (DOCX)

S6 Table Standardized incidence ratio breast cancer (women) and advanced prostate cancer (men). (DOCX)

S7 Table Standardized incidence ratio of obesity-related cancers in patients with a BMI below 30 and 30 and above. Cancers included: liver, kidney, colorectal, gallbladder, pancreas, ovarian, endometrial and advanced prostate cancer, post-menopausal breast cancer and esophageal adenocarcinoma.
(DOCX)

S8 Table Standardized incidence ratio all cancers combined in patients with a BMI below 30 and 30 and above. (DOCX)

S1 Fig Standardized incidence ratio of postmenopausal breast cancer in women. (TIF)

S2 Fig Standardized incidence ratio of advanced prostate cancer in men. (TIF)

S3 Fig Standardized incidence ratio of all and obesity-related cancer stratified to BMI <> 30 in men. (TIF)

S4 Fig Standardized incidence ratio of all and obesity-related cancer stratified to BMI <> 30 in women. (TIF)

S1 File Cancer incidence rate general population. (DOCX)

S2 File SIR data. (XLSX)

## Abbreviations

SIR: Standardized Incidence Ratio
