# Supplementary material for: Sex differences in obesity related cancer incidence in relation to type 2 diabetes diagnosis (ZODIAC-49)
Source: PLoS One. 2018 Jan 25;13(1):e0190870. doi: 10.1371/journal.pone.0190870 (PMC5784905; doi:10.1371/journal.pone.0190870)
Supplement: S1 Table — (DOCX) [file pone.0190870.s001.docx]

S1 Table: Pooled standardized incidence ratio

|  |  | Men and women | | | | Women | | | | Men | | | |
| --- | --- | --- | --- | --- | --- | --- | --- | --- | --- | --- | --- | --- | --- |
|  | Time period (years) | SIR | 95%CI | | | SIR | 95%CI | | | SIR | 95%CI | | |
| All cancer | -5 till 0 | 1.18 | 1.13 | to | 1.23 | 1.40 | 1.31 | to | 1.48 | 1.01 | 0.95 | to | 1.08 |
|  | 0 till 1 | 1.71 | 1.58 | to | 1.85 | 1.82 | 1.60 | to | 2.03 | 1.63 | 1.46 | to | 1.81 |
|  | 1 till 5 | 1.43 | 1.36 | to | 1.49 | 1.67 | 1.56 | to | 1.79 | 1.26 | 1.17 | to | 1.35 |
| Obesity-related cancer | -5 till 0 | 1.42 | 1.33 | to | 1.51 | 1.77 | 1.63 | to | 1.91 | 1.02 | 0.90 | to | 1.13 |
|  | 0 till 1 | 1.80 | 1.59 | to | 2.01 | 2.21 | 1.88 | to | 2.54 | 1.38 | 1.11 | to | 1.64 |
|  | 1 till 5 | 1.67 | 1.56 | to | 1.79 | 2.12 | 1.94 | to | 2.30 | 1.21 | 1.07 | to | 1.35 |
| Non sex-specific obesity-related cancer | -5 till 0 | 1.38 | 1.23 | to | 1.52 | 1.20 | 1.00 | to | 1.40 | 1.52 | 1.31 | to | 1.72 |
|  | 0 till 1 | 2.29 | 1.91 | to | 2.67 | 2.61 | 2.00 | to | 3.23 | 2.04 | 1.56 | to | 2.51 |
|  | 1 till 5 | 1.88 | 1.69 | to | 2.08 | 1.99 | 1.68 | to | 2.29 | 1.80 | 1.54 | to | 2.05 |
