# Supplementary material for: Sex differences in obesity related cancer incidence in relation to type 2 diabetes diagnosis (ZODIAC-49)
Source: PLoS One. 2018 Jan 25;13(1):e0190870. doi: 10.1371/journal.pone.0190870 (PMC5784905; doi:10.1371/journal.pone.0190870)
Supplement: S2 Table — Cancers included: liver, kidney, colorectal, gallbladder, pancreas, ovarian, endometrial and advanced prostate cancer, post-menopausal breast cancer and esophageal adenocarcinoma. (DOCX) [file pone.0190870.s002.docx]

S2 Table: Standardized incidence ratio of obesity-related cancers*

|  | Men and women | | | | Women | | | | Men | | | |
| --- | --- | --- | --- | --- | --- | --- | --- | --- | --- | --- | --- | --- |
| Time period (years) | SIR | 95%CI | | | SIR | 95%CI | | | SIR | 95%CI | | |
| -5 till - 4 | 1.31 | 1.11 | to | 1.52 | 1.68 | 1.37 | to | 2.00 | 0.84 | 0.60 | to | 1.09 |
| -4 till -3 | 1.36 | 1.16 | to | 1.57 | 1.74 | 1.43 | to | 2.05 | 0.91 | 0.67 | to | 1.16 |
| -3 till -2 | 1.40 | 1.20 | to | 1.59 | 1.84 | 1.53 | to | 2.15 | 0.90 | 0.67 | to | 1.13 |
| -2 till -1 | 1.50 | 1.30 | to | 1.69 | 2.06 | 1.74 | to | 2.39 | 0.88 | 0.66 | to | 1.10 |
| -1 till 0 | 1.38 | 1.20 | to | 1.56 | 1.44 | 1.17 | to | 1.70 | 1.32 | 1.06 | to | 1.58 |
| 0 till 1 | 1.80 | 1.59 | to | 2.01 | 2.21 | 1.88 | to | 2.54 | 1.38 | 1.11 | to | 1.64 |
| 1 till 2 | 1.67 | 1.46 | to | 1.88 | 2.16 | 1.82 | to | 2.50 | 1.16 | 0.91 | to | 1.42 |
| 2 till 3 | 1.53 | 1.32 | to | 1.74 | 2.03 | 1.69 | to | 2.37 | 1.02 | 0.77 | to | 1.27 |
| 3 till 4 | 1.69 | 1.46 | to | 1.93 | 2.08 | 1.71 | to | 2.44 | 1.31 | 1.01 | to | 1.60 |
| 4 till 5 | 1.61 | 1.37 | to | 1.85 | 2.08 | 1.70 | to | 2.46 | 1.14 | 0.85 | to | 1.43 |

* cancers included: liver, kidney, colorectal, gallbladder, pancreas, ovarian, endometrial and advanced prostate cancer, post-menopausal breast cancer and esophageal adenocarcinoma.
