# Supplementary material for: Sex differences in obesity related cancer incidence in relation to type 2 diabetes diagnosis (ZODIAC-49)
Source: PLoS One. 2018 Jan 25;13(1):e0190870. doi: 10.1371/journal.pone.0190870 (PMC5784905; doi:10.1371/journal.pone.0190870)
Supplement: S3 Table — Cancers included: liver, kidney, colorectal, gallbladder, pancreas and esophageal adenocarcinoma. (DOCX) [file pone.0190870.s003.docx]

S3 Table: Standardized incidence ratio of obesity-related cancer excluding sex-specific cancers*.

|  | Men and women | | | | Women | | | | Men | | | |
| --- | --- | --- | --- | --- | --- | --- | --- | --- | --- | --- | --- | --- |
| Time period (years) | SIR | 95%CI | | | SIR | 95%CI | | | SIR | 95%CI | | |
| -5 till - 4 | 1.17 | 0.85 | to | 1.48 | 0.98 | 0.55 | to | 1.41 | 1.32 | 0.87 | to | 1.77 |
| -4 till -3 | 1.25 | 0.94 | to | 1.57 | 1.15 | 0.70 | to | 1.60 | 1.34 | 0.90 | to | 1.77 |
| -3 till -2 | 1.27 | 0.97 | to | 1.57 | 0.97 | 0.58 | to | 1.37 | 1.50 | 1.06 | to | 1.94 |
| -2 till -1 | 1.38 | 1.08 | to | 1.69 | 1.64 | 1.14 | to | 2.14 | 1.18 | 0.81 | to | 1.56 |
| -1 till 0 | 1.60 | 1.28 | to | 1.92 | 1.14 | 0.73 | to | 1.54 | 1.96 | 1.49 | to | 2.42 |
| 0 till 1 | 2.29 | 1.91 | to | 2.67 | 2.61 | 2.00 | to | 3.23 | 2.04 | 1.56 | to | 2.51 |
| 1 till 2 | 1.79 | 1.44 | to | 2.14 | 1.95 | 1.39 | to | 2.51 | 1.67 | 1.21 | to | 2.12 |
| 2 till 3 | 1.68 | 1.32 | to | 2.03 | 1.92 | 1.35 | to | 2.50 | 1.49 | 1.04 | to | 1.93 |
| 3 till 4 | 1.89 | 1.49 | to | 2.28 | 1.91 | 1.31 | to | 2.51 | 1.86 | 1.34 | to | 2.39 |
| 4 till 5 | 1.95 | 1.53 | to | 2.37 | 1.98 | 1.34 | to | 2.61 | 1.93 | 1.38 | to | 2.49 |

* cancer included: liver, kidney, colorectal, gallbladder, pancreas and esophageal adenocarcinoma
