# Supplementary material for: Sex differences in obesity related cancer incidence in relation to type 2 diabetes diagnosis (ZODIAC-49)
Source: PLoS One. 2018 Jan 25;13(1):e0190870. doi: 10.1371/journal.pone.0190870 (PMC5784905; doi:10.1371/journal.pone.0190870)
Supplement: S5 Table — (DOCX) [file pone.0190870.s005.docx]

S5 Table: Standardized incidence ratio all cancers combined (excluding pancreas).

|  | Men and women | | | | Women | | | | Men | | | |
| --- | --- | --- | --- | --- | --- | --- | --- | --- | --- | --- | --- | --- |
| Time period (years) | SIR | 95%CI | | | SIR | 95%CI | | | SIR | 95%CI | | |
| -5 till - 4 | 1.06 | 0.94 | to | 1.17 | 1.26 | 1.08 | to | 1.45 | 0.88 | 0.74 | to | 1.03 |
| -4 till -3 | 1.10 | 0.99 | to | 1.22 | 1.32 | 1.13 | to | 1.51 | 0.93 | 0.78 | to | 1.07 |
| -3 till -2 | 1.20 | 1.08 | to | 1.31 | 1.53 | 1.33 | to | 1.73 | 0.93 | 0.79 | to | 1.07 |
| -2 till -1 | 1.24 | 1.13 | to | 1.36 | 1.56 | 1.36 | to | 1.75 | 1.00 | 0.86 | to | 1.14 |
| -1 till 0 | 1.34 | 1.23 | to | 1.46 | 1.35 | 1.16 | to | 1.53 | 1.34 | 1.19 | to | 1.50 |
| 0 till 1 | 1.68 | 1.55 | to | 1.82 | 1.79 | 1.58 | to | 2.00 | 1.60 | 1.43 | to | 1.77 |
| 1 till 2 | 1.49 | 1.35 | to | 1.62 | 1.82 | 1.60 | to | 2.05 | 1.24 | 1.08 | to | 1.40 |
| 2 till 3 | 1.36 | 1.23 | to | 1.50 | 1.57 | 1.35 | to | 1.79 | 1.21 | 1.05 | to | 1.38 |
| 3 till 4 | 1.50 | 1.35 | to | 1.64 | 1.73 | 1.49 | to | 1.98 | 1.33 | 1.15 | to | 1.51 |
| 4 till 5 | 1.45 | 1.30 | to | 1.60 | 1.61 | 1.36 | to | 1.86 | 1.33 | 1.14 | to | 1.52 |
