# Supplementary material for: Sex differences in obesity related cancer incidence in relation to type 2 diabetes diagnosis (ZODIAC-49)
Source: PLoS One. 2018 Jan 25;13(1):e0190870. doi: 10.1371/journal.pone.0190870 (PMC5784905; doi:10.1371/journal.pone.0190870)
Supplement: S6 Table — (DOCX) [file pone.0190870.s006.docx]

S6 Table: Standardized incidence ratio breast cancer (women) and advanced prostate cancer (men).

|  | Women (breast cancer) | | | | Men (advanced prostate cancer) | | | |
| --- | --- | --- | --- | --- | --- | --- | --- | --- |
| Time period (years) | SIR | 95%CI | | | SIR | 95%CI | | |
| -5 till - 4 | 2.19 | 1.68 | to | 2.70 | 0.40 | 0.16 | to | 0.63 |
| -4 till -3 | 2.42 | 1.90 | to | 2.95 | 0.56 | 0.29 | to | 0.82 |
| -3 till -2 | 2.43 | 1.91 | to | 2.94 | 0.40 | 0.19 | to | 0.61 |
| -2 till -1 | 2.37 | 1.87 | to | 2.86 | 0.63 | 0.38 | to | 0.88 |
| -1 till 0 | 1.60 | 1.20 | to | 2.01 | 0.73 | 0.47 | to | 0.99 |
| 0 till 1 | 2.03 | 1.57 | to | 2.48 | 0.77 | 0.50 | to | 1.04 |
| 1 till 2 | 2.12 | 1.63 | to | 2.61 | 0.76 | 0.49 | to | 1.04 |
| 2 till 3 | 2.11 | 1.60 | to | 2.62 | 0.65 | 0.38 | to | 0.92 |
| 3 till 4 | 1.98 | 1.47 | to | 2.50 | 0.83 | 0.52 | to | 1.15 |
| 4 till 5 | 2.11 | 1.54 | to | 2.67 | 0.54 | 0.28 | to | 0.81 |
