# Supplementary material for: Sex differences in obesity related cancer incidence in relation to type 2 diabetes diagnosis (ZODIAC-49)
Source: PLoS One. 2018 Jan 25;13(1):e0190870. doi: 10.1371/journal.pone.0190870 (PMC5784905; doi:10.1371/journal.pone.0190870)
Supplement: S7 Table — Cancers included: liver, kidney, colorectal, gallbladder, pancreas, ovarian, endometrial and advanced prostate cancer, post-menopausal breast cancer and esophageal adenocarcinoma. (DOCX) [file pone.0190870.s007.docx]

S7 Table: Standardized incidence ratio of obesity-related cancers* in patients with a BMI below 30 and 30 and above.

|  |  | Men and women | | | | Women | | | | Men | | | |
| --- | --- | --- | --- | --- | --- | --- | --- | --- | --- | --- | --- | --- | --- |
| BMI | **Time period (years)** | SIR | 95%CI | | | SIR | 95%CI | | | SIR | 95%CI | | |
| < 30 | **0 till 1** | 1.67 | 1.37 | to | 1.97 | 1.92 | 1.44 | to | 2.40 | 1.47 | 1.09 | to | 1.85 |
|  | **1 till 2** | 1.79 | 1.44 | to | 2.13 | 2.49 | 1.89 | to | 3.10 | 1.19 | 0.81 | to | 1.58 |
|  | **2 till 3** | 1.34 | 1.00 | to | 1.67 | 1.81 | 1.24 | to | 2.39 | 0.94 | 0.57 | to | 1.32 |
|  | **3 till 4** | 1.19 | 0.85 | to | 1.54 | 1.15 | 0.65 | to | 1.65 | 1.23 | 0.76 | to | 1.70 |
|  | **4 till 5** | 1.68 | 1.18 | to | 2.18 | 2.05 | 1.23 | to | 2.87 | 1.38 | 0.78 | to | 1.99 |
| >= 30 | **0 till 1** | 1.84 | 1.42 | to | 2.26 | 2.05 | 1.48 | to | 2.62 | 1.53 | 0.93 | to | 2.13 |
|  | **1 till 2** | 1.99 | 1.50 | to | 2.49 | 2.70 | 1.95 | to | 3.44 | 0.95 | 0.41 | to | 1.49 |
|  | **2 till 3** | 1.96 | 1.41 | to | 2.50 | 2.34 | 1.57 | to | 3.12 | 1.38 | 0.66 | to | 2.11 |
|  | **3 till 4** | 1.89 | 1.30 | to | 2.49 | 1.87 | 1.11 | to | 2.64 | 1.92 | 0.98 | to | 2.86 |
|  | **4 till 5** | 1.79 | 1.09 | to | 2.48 | 2.49 | 1.42 | to | 3.55 | 0.72 | 0.01 | to | 1.43 |

*Cancers included: liver, kidney, colorectal, gallbladder, pancreas, ovarian, endometrial and advanced prostate cancer, post-menopausal breast cancer and esophageal adenocarcinoma.
