# Supplementary material for: Sex differences in obesity related cancer incidence in relation to type 2 diabetes diagnosis (ZODIAC-49)
Source: PLoS One. 2018 Jan 25;13(1):e0190870. doi: 10.1371/journal.pone.0190870 (PMC5784905; doi:10.1371/journal.pone.0190870)
Supplement: S8 Table — (DOCX) [file pone.0190870.s008.docx]

S8 Table: Standardized incidence ratio all cancers combined in patients with a BMI below 30 and 30 and above.

|  |  | Men and women | | | | Women | | | | Men | | | |
| --- | --- | --- | --- | --- | --- | --- | --- | --- | --- | --- | --- | --- | --- |
| BMI | **Time period (years)** | SIR | 95%CI | | | SIR | 95%CI | | | SIR | 95%CI | | |
| < 30 | **0 till 1** | 1.75 | 1.54 | to | 1.95 | 1.70 | 1.37 | to | 2.03 | 1.77 | 1.52 | to | 2.03 |
|  | **1 till 2** | 1.65 | 1.43 | to | 1.87 | 2.21 | 1.79 | to | 2.62 | 1.32 | 1.07 | to | 1.56 |
|  | **2 till 3** | 1.27 | 1.06 | to | 1.49 | 1.49 | 1.11 | to | 1.88 | 1.15 | 0.89 | to | 1.40 |
|  | **3 till 4** | 1.26 | 1.03 | to | 1.50 | 1.16 | 0.79 | to | 1.53 | 1.32 | 1.02 | to | 1.62 |
|  | **4 till 5** | 1.34 | 1.05 | to | 1.64 | 1.49 | 0.97 | to | 2.00 | 1.26 | 0.90 | to | 1.62 |
| >= 30 | **0 till 1** | 1.61 | 1.35 | to | 1.87 | 1.67 | 1.30 | to | 2.04 | 1.56 | 1.19 | to | 1.92 |
|  | **1 till 2** | 1.49 | 1.20 | to | 1.77 | 1.88 | 1.43 | to | 2.33 | 1.07 | 0.72 | to | 1.42 |
|  | **2 till 3** | 1.56 | 1.23 | to | 1.89 | 1.76 | 1.27 | to | 2.25 | 1.35 | 0.91 | to | 1.80 |
|  | **3 till 4** | 1.63 | 1.25 | to | 2.00 | 1.56 | 1.05 | to | 2.07 | 1.70 | 1.15 | to | 2.25 |
|  | **4 till 5** | 1.53 | 1.09 | to | 1.97 | 1.77 | 1.12 | to | 2.43 | 1.26 | 0.68 | to | 1.84 |
