# Supplementary figures and images for: Sex differences in obesity related cancer incidence in relation to type 2 diabetes diagnosis (ZODIAC-49)
Source: PLoS One. 2018 Jan 25;13(1):e0190870. doi: 10.1371/journal.pone.0190870 (PMC5784905; doi:10.1371/journal.pone.0190870)

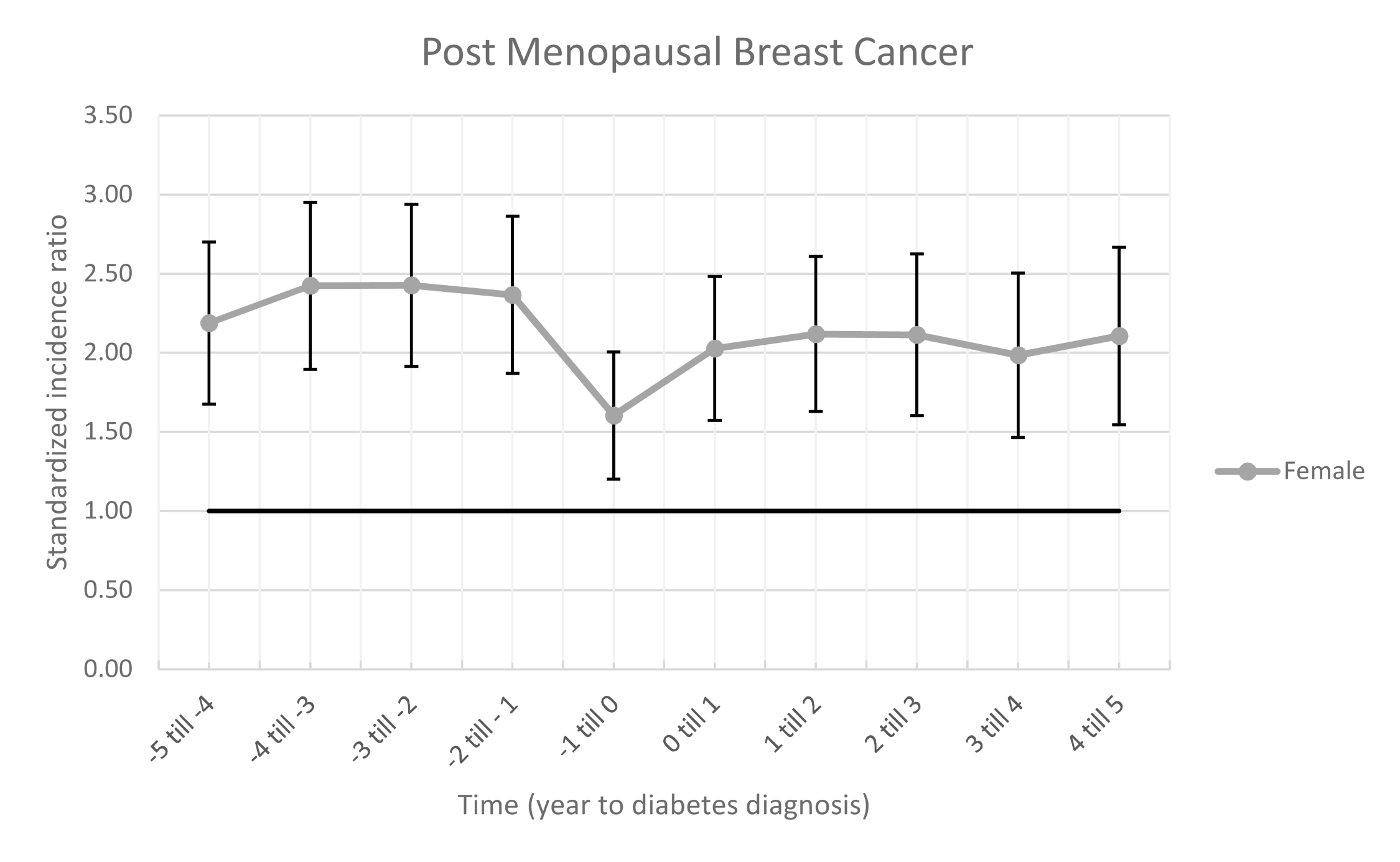

Supplement: S1 Fig — (TIF) [file pone.0190870.s009.tif]

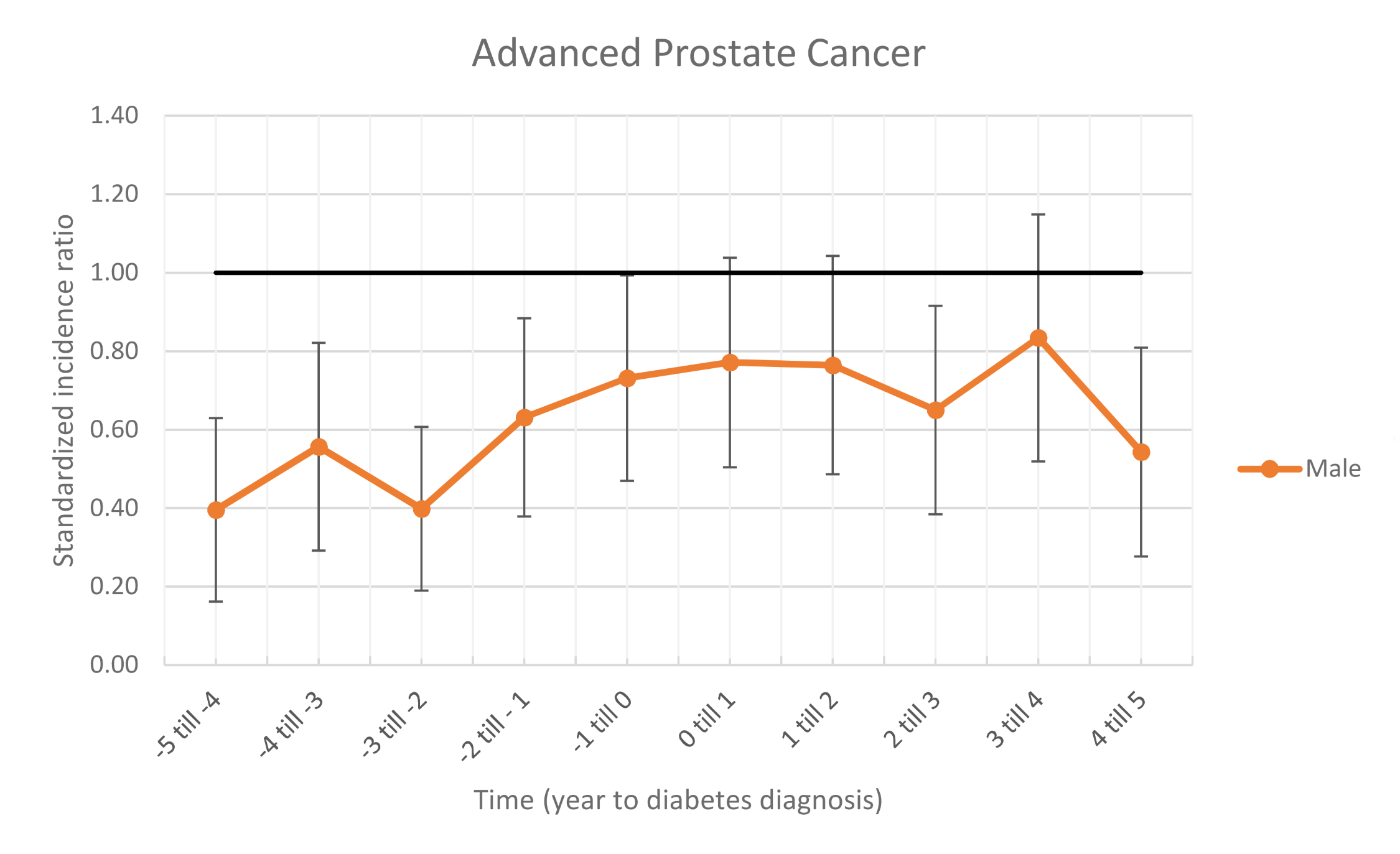

Supplement: S2 Fig — (TIF) [file pone.0190870.s010.tif]

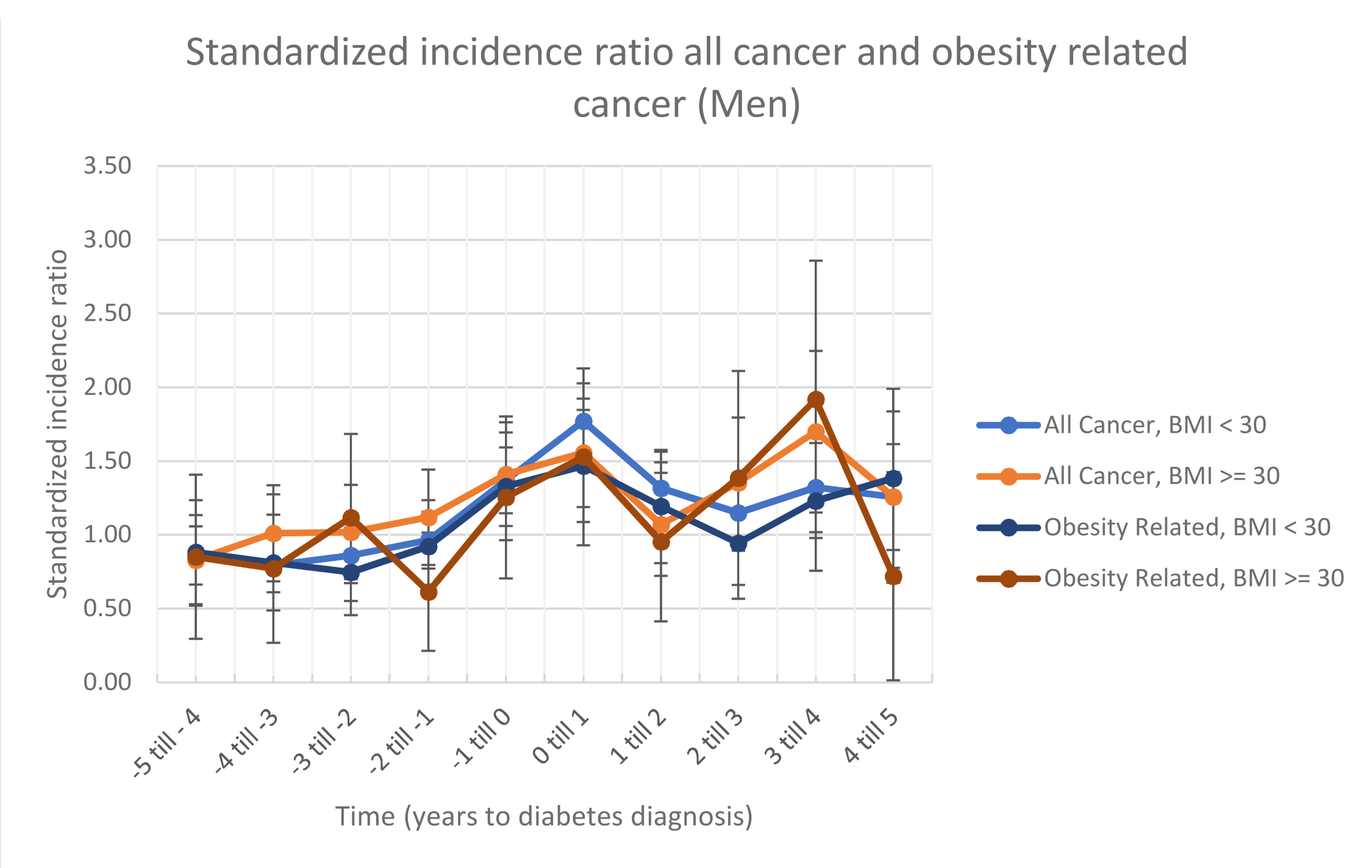

Supplement: S3 Fig — (TIF) [file pone.0190870.s011.tif]

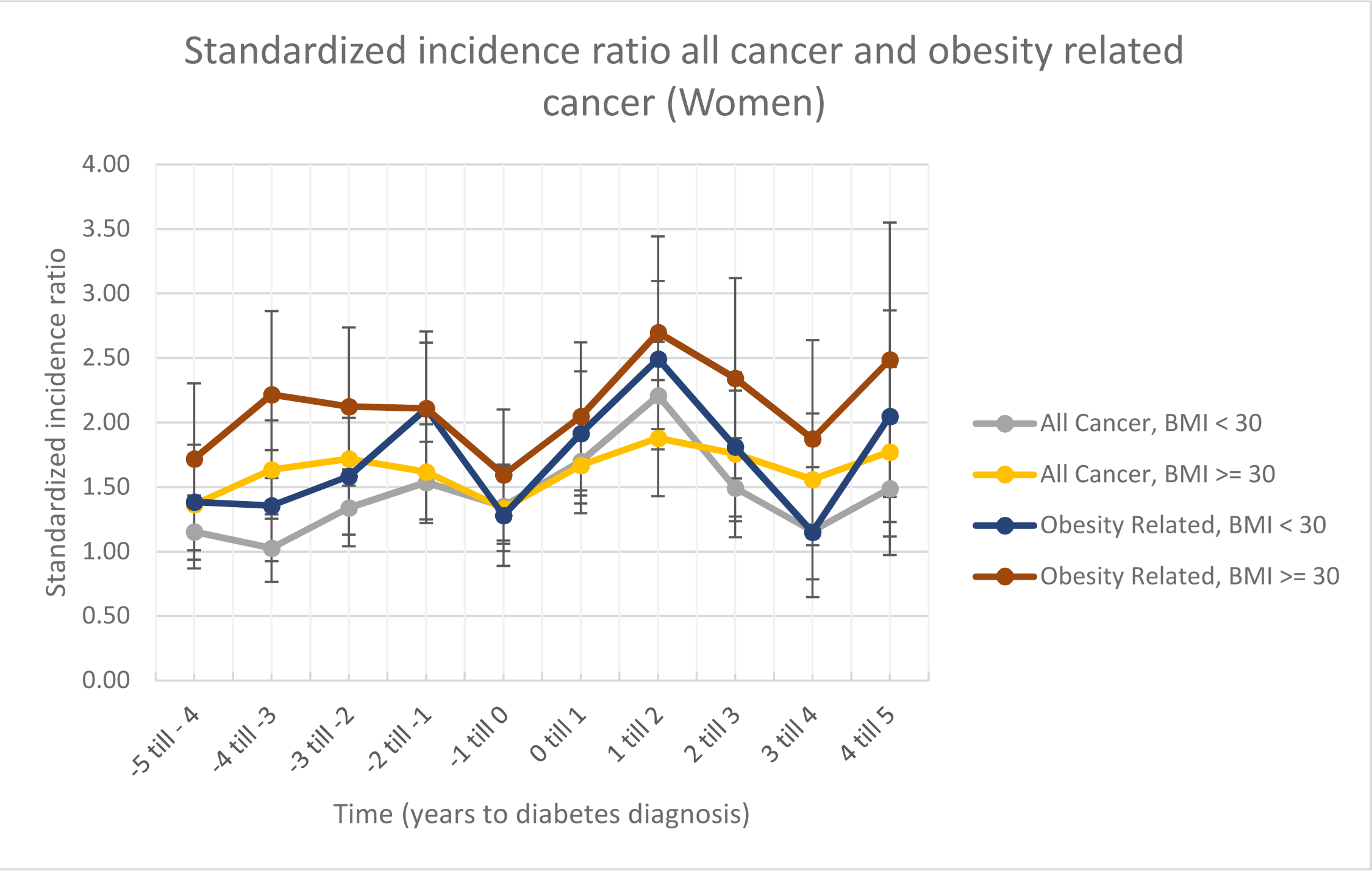

Supplement: S4 Fig — (TIF) [file pone.0190870.s012.tif]
