# Supplementary material for: Sex differences in obesity related cancer incidence in relation to type 2 diabetes diagnosis (ZODIAC-49)
Source: PLoS One. 2018 Jan 25;13(1):e0190870. doi: 10.1371/journal.pone.0190870 (PMC5784905; doi:10.1371/journal.pone.0190870)
Supplement: S1 File — (DOCX) [file pone.0190870.s013.docx]

Table 1: Incidence rate of obesity related cancer in the general population (men)

| Agegroup\ Year | 15-19 | 20-24 | 25-29 | 30-34 | 35-39 | 40-44 | 45-49 | 50-54 | 55-59 | 60-64 | 65-69 | 70-74 | 75-79 | 80-84 | 85-89 | 90-94 | 95+ |
| --- | --- | --- | --- | --- | --- | --- | --- | --- | --- | --- | --- | --- | --- | --- | --- | --- | --- |
| 1989 -1993 | 0.23 | 0.73 | 2.04 | 3.18 | 8.49 | 19.16 | 43.89 | 81.39 | 171.44 | 320.69 | 560.82 | 855.75 | 1184.43 | 1425.28 | 1486.39 | 1210.69 | 816.80 |
| 1994 - 1998 | 0.24 | 0.63 | 1.40 | 3.16 | 7.21 | 18.90 | 42.36 | 99.44 | 215.48 | 412.96 | 719.46 | 1067.13 | 1352.36 | 1519.05 | 1519.24 | 1218.59 | 715.65 |
| 1999-2003 | 0.50 | 0.94 | 1.69 | 3.70 | 7.94 | 20.18 | 45.03 | 114.16 | 246.56 | 488.90 | 792.78 | 1097.17 | 1313.65 | 1369.26 | 1271.48 | 973.76 | 620.93 |
| 2004-2008 | 0.28 | 0.81 | 2.09 | 4.59 | 10.70 | 21.70 | 52.79 | 133.49 | 311.93 | 603.20 | 945.08 | 1241.48 | 1403.50 | 1391.63 | 1286.55 | 983.67 | 684.36 |
| 2009-2012 | 0.30 | 0.82 | 2.67 | 5.11 | 11.88 | 23.66 | 59.87 | 141.41 | 325.71 | 621.24 | 999.29 | 1278.60 | 1437.97 | 1411.72 | 1249.95 | 996.21 | 610.52 |

Table 2: Incidence rate of obesity related cancer in the general population (women)

| Agegroup\ Year | 15-19 | 20-24 | 25-29 | 30-34 | 35-39 | 40-44 | 45-49 | 50-54 | 55-59 | 60-64 | 65-69 | 70-74 | 75-79 | 80-84 | 85-89 | 90-94 | 95+ |
| --- | --- | --- | --- | --- | --- | --- | --- | --- | --- | --- | --- | --- | --- | --- | --- | --- | --- |
| 1989 -1993 | 0.99 | 2.22 | 4.07 | 6.93 | 13.87 | 29.82 | 58.58 | 112.52 | 406.72 | 504.72 | 642.63 | 699.46 | 806.20 | 853.76 | 819.81 | 666.99 | 414.44 |
| 1994 - 1998 | 1.07 | 2.06 | 3.65 | 6.26 | 12.85 | 25.37 | 55.63 | 106.56 | 437.20 | 540.89 | 634.50 | 683.89 | 837.16 | 880.38 | 841.92 | 692.84 | 451.74 |
| 1999-2003 | 1.13 | 2.06 | 3.25 | 6.42 | 12.99 | 22.75 | 54.04 | 106.11 | 466.71 | 551.72 | 645.06 | 786.54 | 813.72 | 899.65 | 847.77 | 656.69 | 369.97 |
| 2004-2008 | 1.02 | 2.13 | 3.75 | 6.99 | 12.53 | 26.41 | 52.99 | 106.14 | 466.82 | 583.90 | 704.54 | 802.40 | 776.90 | 947.51 | 962.70 | 782.37 | 460.87 |
| 2009-2012 | 1.21 | 2.46 | 4.15 | 7.58 | 12.46 | 26.33 | 51.58 | 99.29 | 463.85 | 606.32 | 754.11 | 844.14 | 801.67 | 937.56 | 961.01 | 770.13 | 506.26 |
